# A New Process for the Synthesis of Budesonide 21-Phosphate and Evaluation in a Murine Model of Inflammation

**DOI:** 10.3390/molecules29184514

**Published:** 2024-09-23

**Authors:** Angela Corvino, Elisabetta Granato, Antonia Scognamiglio, Ferdinando Fiorino, Francesco Frecentese, Elisa Magli, Elisa Perissutti, Vincenzo Santagada, Giuseppe Cirino, Ida Cerqua, Rocco Pavese, Antonio Petti, Francesca Pavese, Francesco Petti, Fiorentina Roviezzo, Beatrice Severino, Giuseppe Caliendo

**Affiliations:** 1 Department of Pharmacy, School of Medicine, University of Naples Federico II, Via D. Montesano, 49, 80131 Napoli, Italy; angela.corvino@unina.it (A.C.); elisabetta.granato@unina.it (E.G.); antonia.scognamiglio@unina.it (A.S.); fefiorin@unina.it (F.F.); frecente@unina.it (F.F.); perissut@unina.it (E.P.); santagad@unina.it (V.S.); cirino@unina.it (G.C.); ida.cerqua@unina.it (I.C.); roviezzo@unina.it (F.R.); caliendo@unina.it (G.C.); 2 Department of Public Health, School of Medicine, University of Naples Federico II, Via Pansini, 5, 80131 Napoli, Italy; elisa.magli@unina.it; 3 Genetic S.p.A., Via della Monica, n. 26, 84083 Castel San Giorgio, Italy; rocco.pavese@geneticspa.com (R.P.); a.petti64@gmail.com (A.P.); francesca.pavese@geneticspa.com (F.P.); pettifrancesco95@gmail.com (F.P.)

**Keywords:** anti-inflammatory drugs, inhaled corticosteroids, inflammation, phosphorylation

## Abstract

In this study, a new and straightforward process for the preparation of budesonide 21-phosphate (Bud-21P) and its disodium salt (Bud-21P-Na2) is described. The method results in a yield comparable to those obtained by diphosphoryl chloride, but it is more manageable, less expensive, and safer. The new compounds are characterized by better water solubility compared to the parent compound. Moreover, they have been evaluated for their anti-inflammatory activity and the obtained results clearly evidence that Bud-21P and Bud-21P-Na2 retained anti-inflammatory activity like the parent compound budesonide (Bud) in mice with cutaneous induced edema.

## 1. Introduction

**Figure 1:**
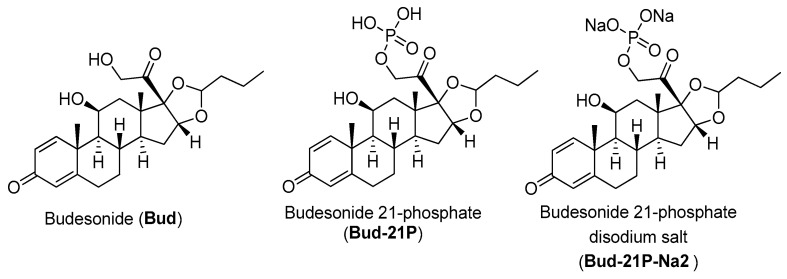
Budesonide, budesonide 21-phosphate, and budesonide 21-phosphate disodium salt structures.

Budesonide (Bud) (IUPAC name (6aR,6bS,7S,8aS,8bS,11aR,12aS,12bS)-7-hydroxy-8b-(2-hydroxyacetyl)-6a,8a-dimethyl-10-propyl-6a,6b,7,8,8a,8b,11a,12,12a,12b-decahydro-1H-naphtho [2’,1’:4,5]indeno [1,2-d][1,3]dioxol-4(2H)-one) is a glucocorticoid steroid, belonging to inhaled corticosteroids (ICSs) [1]. These latter represent, by far, the most effective therapeutic tools used in the treatment of asthma, chronic obstructive pulmonary disease (COPD), noninfectious rhinitis, and Crohn disease (Figure 1).

Budesonide, with a logP of 3.2, is virtually insoluble in water (28 μg/mL) while it is readily soluble in alcohols [2]. When budesonide solutions are prepared using water–alcohol mixtures as solubilizers, the obtained hydroalcoholic solutions are unstable, and a great percentage of budesonide decomposes within a short time. Moreover, when budesonide formulations are prepared as aqueous suspensions, the compound tends to deposit as a solid phase at the bottom of the container, thus requiring chemical additives or vigorous stirring. These are the reasons that make budesonide not suitable to be delivered by an electric nebulizer.

The 21-phosphate primary esters of several corticosteroids have been prepared for prodrug synthesis to increase the solubility of poorly water-soluble orally and parenterally administered drugs [3]. The so-obtained prodrugs are used as active ingredients for several pharmaceutical compositions. The phosphate prodrug is acted upon by endogenous phosphatase enzymes like alkaline phosphatases, present in plasma, and on the apical membrane of enterocytes’ brush border, causing the release of the parent drug molecule by cleaving the phosphate prodrug [4].

Several synthetic approaches for the preparation of phosphate esters of biologically active molecules have been reported. Some of these have as their drawbacks the competitive formation of tri-substituted esters or the formation of the corresponding pyrophosphates. Still, others require the use of a large excess of alcohol or phosphorylation reagents. To avoid such side products, organic triphosphates containing two cleavable ester residues have been used. Several authors have described methodologies involving the use of phosphorus trichloride, diethylchlorophosphite, and phosphoryl chloride to obtain the desired phosphate esters [5,6,7]. Some of these reagents are not at all suitable for acid-sensitive alcohols.

Budesonide 21-phosphate (Bud-21P) (IUPAC name 2-((6aR,6bS,7S,8aS,8bS,11aR,12aS,12bS)-7-hydroxy-6a,8a-dimethyl-4-oxo-10-propyl-2,4,6a,6b,7,8,8a,8b,11a,12,12a,12b-dodecahydro-1H-naphtho [2’,1’:4,5]indeno [1,2-d][1,3]dioxol-8b-yl)-2-oxoethyl dihydrogen phosphate) (Figure 1) has already been synthesized and used as a linker for the targeted delivery of antibody–drug conjugates [8]. Its disodium salt (Bud-21 P-Na2) (IUPAC name sodium 2-((6aR,6bS,7S,8aS,8bS,11aR,12aS,12bS)-7-hydroxy-6a,8a-dimethyl-4-oxo-10-propyl-2,4,6a,6b,7,8,8a,8b,11a,12,12a,12b-dodecahydro-1H-naphtho [2’,1’:4,5]indeno [1,2-d][1,3]dioxol-8b-yl)-2-oxoethyl phosphate), instead, has been used for the preparation of liposomal glucocorticoids studied as antitumor agents [9,10]. Nonetheless, their anti-inflammatory properties have not been detailed so far.

Bud-21P is customarily prepared by the reaction of budesonide with diphosphoryl chloride in THF at −40 °C. To identify a novel and efficient process for the preparation of Bud-21P and its disodium salt that provides the products with good yields and is suitable for the industrial scale, several phosphorylation methods have been examined. Moreover, the anti-inflammatory properties of the obtained compounds have been evaluated to verify if they could be useful in the prevention and/or treatment of acute or chronic conditions, such as respiratory, cutaneous, or neurodegenerative inflammatory pathologies. The obtained results showed that Bud-21P-Na2, in conjunction with a higher water solubility level, showed beneficial effects in mice with cutaneous induced edema, comparable to the parent budesonide.

## 2. Results and Discussion

### 2.1. Chemical Synthesis

The phosphorylation of budesonide is, usually, performed using diphosphoryl chloride as a phosphorylating agent. The latter reacts with water to produce HCl and H_3_PO_4_, making it necessary to preserve both the reagent and the reaction mixture from contact with moisture, requiring more careful preparation of the reaction mixture and, therefore, more expensive chemical procedures. In addition, diphosphoryl chloride gives rise to a strongly exothermic reaction that necessitates operating temperatures of −40 °C. Maintaining this condition requires experimental methodologies that are costly both in terms of energy and specialized personnel employed.

**Scheme 1:**
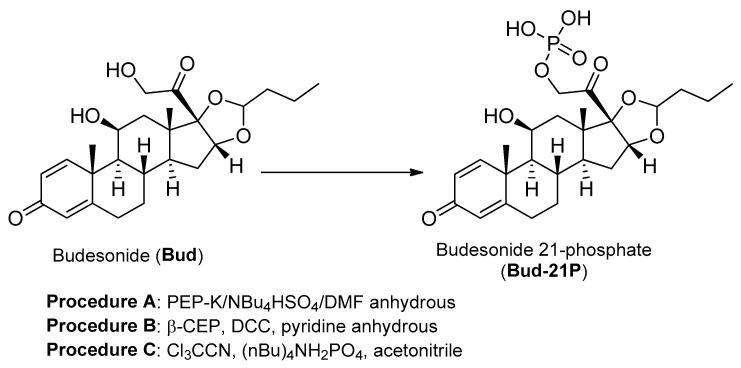
Preparation of budesonide 21-phosphate.

With the aim of developing a more industrially viable procedure, the following methods were evaluated for overall efficiency (Scheme 1): phosphorylation with (A) tetrabutylammonium hydrogen sulfate (TBAHS) and phosphoenolpyruvic acid monopotassium salt (PEP-K) [11], (B) beta-cyanoethyl phosphate in the presence of N,N’-dicyclohexylcarbodiimide (DCC) [12], and (C) tetrabutylammonium hydrogen phosphate and trichloroacetonitrile [13].

Among the protocols evaluated, procedure A (TBAHS/PEP-K), conducted under conventional and microwave heating, afforded the desired compound with a yield in the range of 30–35%. Procedure B (β-CEP/DCC) did not produce the desired compound at room temperature, which caused the decomposition of the starting material when performed under heating, in both conventional and microwave-assisted heating.

Finally, the one-pot procedure using tetrabutylammonium hydrogen phosphate and trichloroacetonitrile (procedure C) provided the budesonide 21-phosphate with an improved yield (83%). The synthetic procedure is a notable improvement with respect to the prior art because the reaction takes place under very mild conditions and at room temperature. Therefore, it is more manageable, less expensive, and safer. Furthermore, under such experimental conditions, no strong acids are produced.

The following conversion to the corresponding disodium salt was realized by titration with 2N NaOH, affording the desired compound with a good yield (79%).

Bud-21P-Na2 has a much higher water solubility than Bud and Bud-21P. Its solubility can be defined as “freely soluble in water (100–1000 mg/mL)” and is equal to 110 mg/mL. At the concentration of use (0.25 mg/mL–4.0 mg/mL), it is rapidly soluble and remains stable at room temperature for long periods of time (12 months) without yellowing or precipitating.

### 2.2. Pharmacological Evaluation

#### 2.2.1. Effect of Bud and Derived Compounds on Inflammatory Reaction

**Figure 2:**
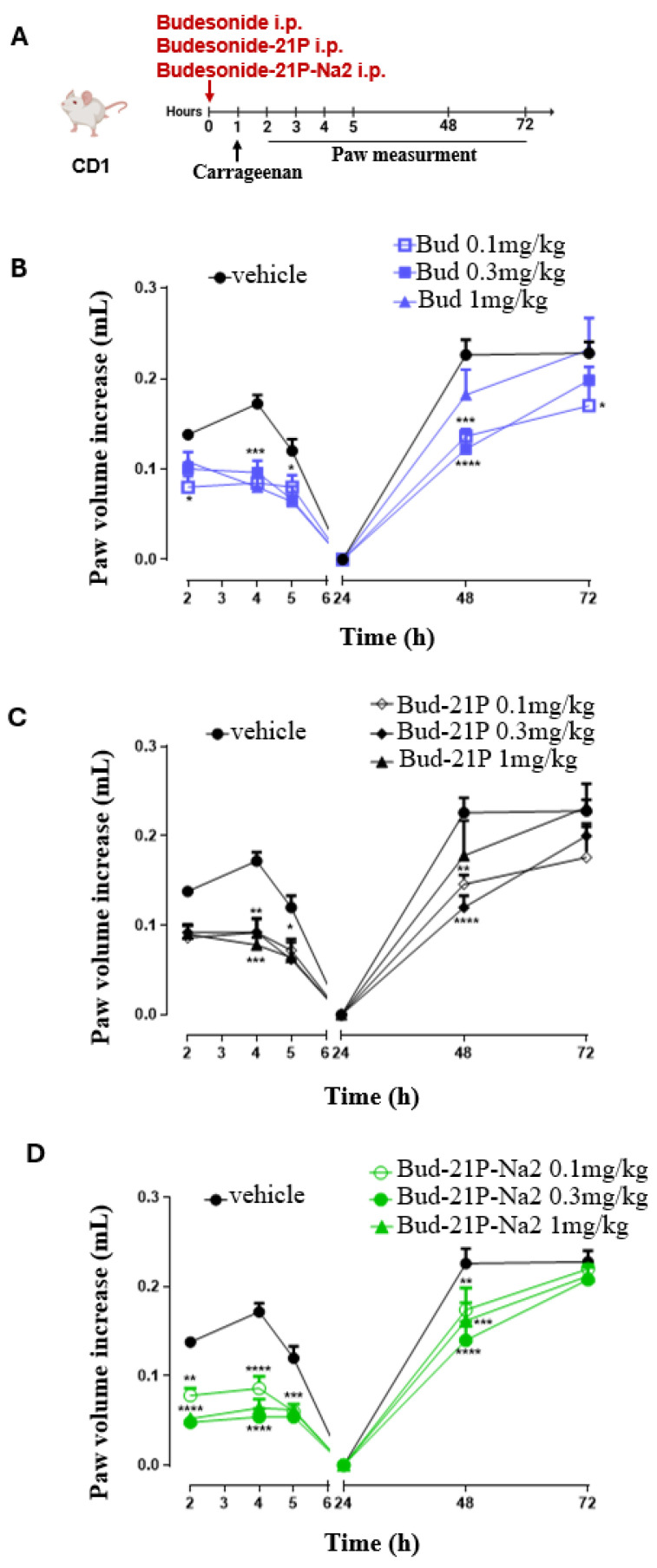
Effect of corticosteroids on carrageenan-induced paw edema: (**A**) paw volume was induced by sub-plantar injection of carrageenan (1%) and edema was measured using a hydroplethismometer immediately before the sub-plantar injection and at 1, 2, 4, 5, 48, and 72 h. The animals were pre-treated (i.p.) 60 min before edema induction with Bud at doses of 0.1, 0.3, and 1 mg/kg (**B**), Bud-21P at doses of 0.1, 0.3, and 1 mg/kg (**C**), and Bud-21P-Na2 at doses of 0.1, 0.3, and 1 mg/kg (**D**); * *p* < 0.05, ** *p* < 0.01, *** *p* < 0.001, and **** p < 0.0001 vs. vehicle-treated group. The data were analyzed by a two-way ANOVA followed by Bonferroni’s test.

Administration of carrageenan induces a significant increase in the paw volume already after two hours, reaching the maximal response at 4 h. The obtained results show a difference in terms of pharmacological activity between the tested drugs in the first phase (6 h). Bud-21P-Na2 shows a dose-related anti-inflammatory effect (Figure 2C), inhibiting edema formation at a dose of 0.1 mg/kg by 50%, and the maximum inhibition achieved is 70% with either 0.3 or 1 mg/kg. This effect is already present at the onset of the edema and lasts throughout the first phase.

**Figure 3:**
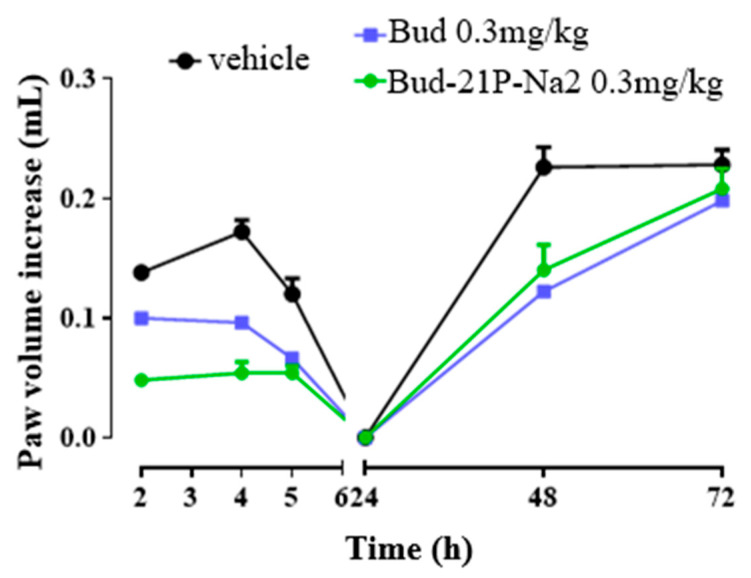
Comparative effects of budesonide 21-phosphate disodium salt and budesonide at a dose of 0.3 mg/kg on carrageenan-induced paw edema. Edema was assessed after the sub-plantar injection of carrageenan. The animals were pre-treated (i.p.) with corticosteroids 60 min before edema induction.

Moreover, Bud induces a significant inhibition in edema development. It can be noted that the maximum inhibition is about 50% but it is not dose-related (Figure 3). At a dose of 0.3 mg/kg, Bud-21P-Na2 shows a more rapid onset of the effect since a significant inhibition is already present after 2 h and remains constant throughout the first phase. Conversely, Bud, at the same dose, reaches the same level of inhibition of Bud-21P-Na2 only after 6 h (Figure 3). After an apparent resolution of the edema, 6 h after the administration of carrageenan, a second phase develops with a more prolonged and lasting reaction that reaches a plateau after 72 h. Although the drugs were administered only when the inflammatory reaction was induced, their pharmacological action is also reflected in the second phase. All tested drugs significantly inhibit edema with a similar profile (Figure 2).

#### 2.2.2. Effect of Bud and Derived Compounds on Allergic Inflammatory Reaction

**Figure 4:**
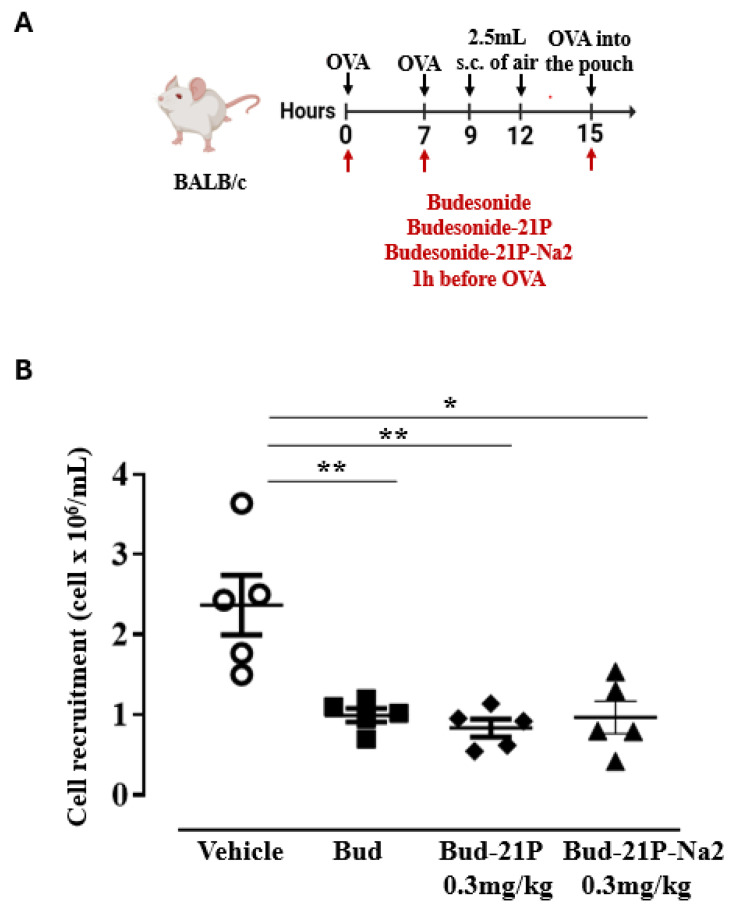
Cell infiltration was quantified at 24 h after the injection of 10 μg of OVA into the dorsal air pouch of sensitized mice; the compounds were administered 60 min before OVA injection into the air pouch (**A**); and (**B**) total cell counts were performed following Trypan blue staining. * *p* < 0.05, ** *p* < 0.01 vs. vehicle-treated group, analyzed by unpaired Student’s *t* test.

The administration of ovalbumin into the air pouch of sensitized mice induces a significant cell infiltrate, which reaches its maximum after 24 h. Administration of all compounds significantly inhibits cell recruitment as shown in Figure 4.

## 3. Materials and Methods

### 3.1. Chemistry

All of the commercial products were purchased from Merck (Merck Life Science, Milano, Italy). Reactions were stirred at 400 rpm by a Heidolph MR Hei-Standard magnetic stirrer. Solutions were concentrated with a Buchi R-114 rotary evaporator (Buchi Italia, Cornaredo, Italy) at a low pressure. All reactions were followed by TLC carried out on Merck silica gel 60 F254 plates (Merck Life Science, Milano, Italy) with a fluorescent indicator on the plates and were visualized with UV light (254 nm). Melting points, determined using a Buchi Melting Point B-540 instrument (Buchi Italia, Cornaredo, Italy), are uncorrected and represent values obtained on recrystallized or chromatographically purified material. NMR spectra of 1H (500 MHz) and 13C (125 MHz) were recorded on an Agilent INOVA spectrometer (Varian Inc., Palo Alto, CA, USA); chemical shifts were referenced to the residual solvent signal (CD_3_OD: δH = 3.31, δC = 49.0). ESI-MS spectra were recorded on an LTQ Orbitrap XL™ Fourier-transform mass spectrometer (FTMS) (Thermo Fisher, San José, CA, USA) equipped with an ESI ION MAX™ (Thermo Fisher, San José, CA, USA). IR spectra were recorded on a Thermo Nicolet 5700 FT-IR spectrometer (Thermo Fisher, San José, CA, USA). NMR (Figures S1–S4), ESI-MS (Figure S5), and IR (Figures S6 and S7) spectra are reported in Supplementary Materials.

#### 3.1.1. Preparation of Budesonide 21-Phosphate (Bud-21P)

##### Catalytic Chemoselective O-Phosphorylation Using Tetrabutylammonium Hydrogen Sulfate (TBAHS) and Phosphoenolpyruvic Acid Monopotassium Salt (PEP-K)

(a) Under conventional heating

A nitrogen-flushed flask equipped with a magnetic stirrer bar was charged with budesonide (42 mg, 0.097 mmol, 1.0 equiv.), tetrabutylammonium hydrogen sulfate (20 mg, 0.058 mmol, 0.60 equiv.), and phosphoenolpyruvate monopotassium salt (200 mg, 0.97 mmol, 10 equiv.). To the reaction mixture, N,N-dimethylformamide (5 mL, 0.20 M) was added at r.t., and the mixture was warmed to 100 °C. The reaction mixture was monitored by TLC using CHCl_3_/MeOH/H_2_O (5/4/1) as an eluent mixture. After stirring for 5 h, the reaction mixture was cooled to r.t. and dried, and the residue was purified by preparative column chromatography, using CHCl_3_/MeOH/CH_3_COOH (8/2/0.3) to give 15 mg of budesonide phosphate (yield 30%).

(b) Under microwave heating

The synthesis was performed using a microwave oven specially designed for organic chemistry (ETHOS 1600, Milestone) (FKV, Bergamo, Italy). The experimental conditions used in the microwave-assisted synthesis were like those used in conventional heating with the same stoichiometric ratios. The reaction was performed using an appropriate microwave program, composed of ramping and holding steps, monitoring the temperature of the mixture directly by a microwave-transparent fluoroptic probe (FKV, Bergamo, Italy).

Budesonide (20 mg, 0.046 mmol, 1.0 equiv.), tetrabutylammonium hydrogen sulfate (9 mg, 0.028 mmol, 0.60 equiv.), and phosphoenolpyruvate monopotassium salt (95 mg, 0.46 mmol, 10 equiv.) dissolved in N,N-dimethylformamide (5 mL) were placed in standard Pyrex glassware with a reflux condenser fitted through the roof of the microwave cavity, which was equipped with a temperature control unit, and irradiated according to the following parameters: initial power, 500 W; initial time, 5 min (ramping); final power, 500 W; T, 100 °C; and reaction time, 4 h. The reaction was monitored by TLC using CHCl_3_/MeOH/H_2_O (5/4/1) as the eluent, then the mixture was cooled to r.t. and dried, and the residue was purified by preparative column chromatography, using CHCl_3_/MeOH/CH_3_COOH (8/2/0.3) to give 8.2 mg of budesonide phosphate (yield 35%).

##### Phosphorylation by Tetrabutylammonium Hydrogen Phosphate and Trichloroacetonitrile

To a solution of budesonide (200 mg, 0.46 mmol) in acetonitrile (1 mL), trichloroacetonitrile (220 mL, 2.20 mmol) was added, followed by dropwise addition of tetrabutylammonium dihydrogen phosphate (625 mg, 1.84 mmol) in acetonitrile (2 mL). The reaction mixture was stirred at room temperature for 24 h and monitored by TLC using CHCl_3_/MeOH/CH_3_COOH (8/2/0.3) as the eluent. Then, it was treated with 1 N NaOH and extracted with ethyl acetate. The aqueous phase was made acidic using a 1 N HCl solution and extracted several times with ethyl acetate. The combined organic phases were washed with brine, dried over sodium sulfate, and concentrated to give 195 mg of budesonide 21-phosphate (yield 83%). M.P. 219–221 °C LR-MS (ES) (M + H)^+^: calcd, 510.5; found, 511.2.

1H NMR (500 MHz, CD_3_OD-d_4_) δ 7.47 (d, *J* = 10.1 Hz, 1H), 6.26 (d, *J* = 10.1 Hz, 1H), 6.01 (s, 1H), 5.18 (dd, *J* = 13.1, 6.2 Hz, 1H), 4.96–4.83 (m, 2H), 4.72–4.62 (m, 2H), 4.41 (d, *J* = 3.5 Hz, 1H), 2.64 (dt, *J* = 13.0, 6.7 Hz, 1H), 2.37 (d, *J* = 11.0 Hz, 1H), 2.24–2.09 (m, 3H), 1.94 (dd, *J* = 17.8, 9.7 Hz, 1H), 1.70 (dd, *J* = 14.1, 6.6 Hz, 1H), 1.60 (dd, *J* = 12.1, 7.0 Hz, 3H), 1.51–1.39 (m, 4H), 1.04–0.89 (m, 7H). 13C NMR (126 MHz, CD_3_OD-d_4_): δ 210.89, 209.55, 188.85, 174.28, 159.86, 127.84, 122.55, 109.41, 105.45, 99.88, 98.97, 84.01, 82.92, 70.53, 70.48, 69.96, 69.68, 57.17, 57.08, 54.22, 51.33, 47.07, 45.98, 45.94, 41.34, 40.97, 38.27, 36.17, 35.50, 35.35, 34.34, 33.83, 33.01, 32.47, 31.75, 21.55, 18.44, 17.98, 17.82, 17.54, 14.40, 14.26.

#### 3.1.2. Preparation of Budesonide 21-Phosphate Disodium Salt (Bud-21P-Na2)

Budesonide 21-phosphate (100 mg, 0.196 mmol) was suspended in water (10 mL) and titrated with 2N NaOH to pH 7.94, resulting in a completely clear solution. Then, the solvent was removed, and the residue was treated with methanol (5 mL), keeping the suspension at the boiling point of the solvent for 30 min. After cooling, the insoluble solid was filtered off, and the solvent was removed in vacuo. The residue was then treated with diethyl ether, affording budesonide 21-phosphate disodium salt as a white solid (86 mg, yield 79%), M.P. 245–246 °C.

1H NMR (500 MHz, CD_3_OD-d_4_) δ 7.47 (d, *J* = 10.1 Hz, 1H), 6.26 (d, *J* = 10.1 Hz, 1H), 6.01 (s, 1H), 5.18 (dd, *J* = 13.1, 6.2 Hz, 1H), 4.96–4.83 (m, 2H), 4.72–4.62 (m, 2H), 4.41 (d, *J* = 3.5 Hz, 1H), 2.64 (dt, *J* = 13.0, 6.7 Hz, 1H), 2.37 (d, *J* = 11.0 Hz, 1H), 2.24–2.09 (m, 3H), 1.94 (dd, *J* = 17.8, 9.7 Hz, 1H), 1.70 (dd, *J* = 14.1, 6.6 Hz, 1H), 1.60 (dd, *J* = 12.1, 7.0 Hz, 3H), 1.51–1.39 (m, 4H), 1.04–0.89 (m, 7H). 13C NMR (126 MHz, CD_3_OD-d_4_): δ 210.89, 209.55, 188.85, 174.28, 159.86, 127.84, 122.55, 109.41, 105.45, 99.88, 98.97, 84.01, 82.92, 70.53, 70.48, 69.96, 69.68, 57.17, 57.08, 54.22, 51.33, 47.07, 45.98, 45.94, 41.34, 40.97, 38.27, 36.17, 35.50, 35.35, 34.34, 33.83, 33.01, 32.47, 31.75, 21.55, 18.44, 17.98, 17.82, 17.54, 14.40, 14.26.

### 3.2. Pharmacology

#### 3.2.1. Animals

Female BALB/c mice and male CD-1 mice were purchased from Charles River (Lecco, Italy). The animals were housed in a controlled environment and provided with standard rodent chow and water. All the animals were anesthetized before being humanely sacrificed by way of carbon dioxide euthanasia. All efforts were made to minimize the number of animals used and their suffering. Mice were blindly randomized, and all experimental procedures and protocols followed national laws and policies approved by the Italian Ministry of Health (Directive 2010/63/UE).

#### 3.2.2. Test Substances and Reagents

A volume of 100 μL of a solution of test compounds Bud, Bud-21P, and Bud-21P-Na2 was administered intraperitoneally 1 h before the induction of the inflammatory reaction. Doses of 0.1, 0.3, and 1 mg/kg were tested. In the air pouch induced by ovalbumin in sensitized mice, Bud and Bud-21P-Na2 (0.3 mg/kg) were tested. Both compounds were administered in a final volume of 100 µL into the air pocket 1h before the induction of the inflammatory reaction.

Chicken egg-white ovalbumin (OVA; grade V, cat. A5503, Sigma Chemical Co., St. Louis, MO, USA) was dissolved in a sterile phosphate-buffered saline (PBS) solution (250 µg/mL), and Al(OH)_3_ was added (at 13 mg/mL). This mix was used to induce the allergic sensitization of the animals by subcutaneous injection. OVA was dissolved at 1% in the sterile PBS solution, and this solution was nebulized (as the immunological challenges).

#### 3.2.3. Paw Edema

Carrageenan edema in the paw of the CD-1 mice involves the sub-plantar administration of 50 µL of 1% carrageenan and the measurement of the increase in the volume of the paw by using a hydroplethysmometer. This experimental model has a biphasic development [14]. The first phase, which is acute, is sustained by an increase in permeability accompanied by the infiltration of neutrophils, which develops and resolves between 4 and 5 h. The second phase occurs after 24 h and is characterized by macrophage infiltration and has the characteristics of sub-chronic inflammation, which reaches its maximum after 72 h and resolves after 5 days. Bud and Bud-21P-Na2 were tested. Both compounds were administered in a volume of 100 µL intraperitoneally 1 h before the induction of the inflammatory reaction. Doses of 0.1, 0.3, and 1 mg/kg were tested.

#### 3.2.4. Air Pouch

BALB/c female mice were injected with 0.4 mL s.c. of a suspension containing 100 μg of OVA absorbed into 3.3 mg of aluminum hydroxide gel on days 0 and 7. Mice, sensitized as described above, received, on days 9 and 12 on the shaved dorsal surface, 2.5 mL s.c. of air to initiate the development of the air pouches as described previously. On day 15 (6 days after the first air injection), animals were challenged by injection into the air- pouch with 0.4 mL of sterile saline alone or containing 10 μg OVA. Budesonide and both the disodium salt and phosphate were tested (0.3 mg/kg); drugs or vehicles were administered i.p. in a final volume of 100 μL 60 min before each OVA administration. After OVA or saline injection into the air pouch, mice were sacrificed by exposition to CO_2_. Air pouches were washed with 1 mL phosphate-buffered saline (pH = 7.4). Lavage fluids were centrifuged at 300× *g* for 10 min at 4 °C. Cell pellets were resuspended in phosphate-buffered saline, and total cell counts were performed following Trypan blue staining. At 24 h, the cell infiltrate mainly constituted polymorphonuclear cells (PMNs), including a significant proportion of mast cells and eosinophils [15]. Bud and Bud-21P-Na2 (0.3 mg/kg) were tested. Both compounds were administered in a final volume of 100 µL into the air pocket 1h before the induction of the inflammatory reaction.

#### 3.2.5. Statistical Analysis

Data are expressed as the arithmetic mean ± SEM from n individual animals. Statistical analysis of the data was carried out using Software GraphPad Prism v5.01. The results were analyzed using a one-way ANOVA, followed by Dunnett’s multiple comparison test; differences between group means with *p* < 0.05 values were considered significant.

## 4. Conclusions

Here, we describe a new process for the conversion of budesonide (Bud), a well-known anti-inflammatory drug, to the corresponding 21-phosphate derivative (Bud-21P) and the subsequent preparation of its disodium salt (Bud-21P-Na2). Three phosphorylation methods have been evaluated, and conventional and microwave-heated conditions have been compared. The one-pot procedure using tetrabutylammonium hydrogen phosphate and trichloroacetonitrile allowed us to convert Bud to Bud-21P with a very good yield (83%). The described method is a straightforward approach to the synthesis of Bud-21P, resulting in a yield comparable to those obtained by diphosphoryl chloride, but is more manageable, less expensive, and safer. As expected, Bud-21P and Bud 21P-Na2 exhibit better water solubility than budesonide, with the disodium salt being defined as “freely soluble in water (100–1000 mg/mL)”. Finally, biological evaluation in rat paw carrageenan edema, a well-known murine model of inflammation, shows that Bud-21P and Bud 21P-Na2 retain anti-inflammatory activity comparable to that of the starting compound. At this point in our research, we hypothesize that the mechanism of action of Bud-21P is the same as other 21-phosphate steroids. Therefore, it might exert its anti-inflammatory activity as a result of the actions of enzymes with phosphatase activity, e.g., alkaline phosphatase, which releases the starting corticosteroid. Further experiments are needed to confirm this hypothesis. 

## Data Availability

Data are contained within the article and Supplementary Materials.

## Supplementary Materials

The following supporting information can be downloaded at https://www.mdpi.com/article/10.3390/molecules29184514/s1: Figure S1: 1H-NMR (500 MHz; CD3OD-d4) spectrum of Budesonide 21-phosphate; Figure S2: 13C-NMR (126 MHz; CD3OD-d4) spectrum of Budesonide 21-phosphate; Figure S3: 1H-NMR (500 MHz; CD3OD-d4) spectrum of Budesonide 21-phosphate disodium salt; Figure S4: 13C-NMR (126 MHz; CD3OD-d4) spectrum of Budesonide 21-phosphate disodium salt; Figure S5: ESI-MS spectrum of Budesonide 21-phosphate; Figure S6: FT-IR spectrum of Budesonide 21-phosphate; Figure S7: FT-IR spectrum of Budesonide 21-phosphate disodium salt.
