# Supplementary material for: A New Process for the Synthesis of Budesonide 21-Phosphate and Evaluation in a Murine Model of Inflammation
Source: Molecules. 2024 Sep 23;29(18):4514. doi: 10.3390/molecules29184514 (PMC11434587; doi:10.3390/molecules29184514)
Supplement: Supplementary file 1 [file molecules-29-04514-s001.zip › molecules-3205823-supplementary.pdf]

A New Process for the Synthesis of Budesonide 21-Phosphate and Evaluation in a Murine Model of Inflammation

Angela Corvino,[1] Elisabetta Granato,[1] Antonia Scognamiglio,[1] Ferdinando Fiorino,[1] Francesco Frecentese,[1] Elisa Magli,[2] Elisa Perissutti,[1] Vincenzo Santagada,[1] Giuseppe Cirino,[1] Ida Cerqua,[1] Rocco Pavese,[3] Antonio Petti,[3] Francesca Pavese,[3] Francesco Petti,[3] Fiorentina Roviezzo,[1] Beatrice Severino,[1]* and Giuseppe Caliendo[1]

[1]Department of Pharmacy, School of Medicine, University of Naples Federico II, Via D. Montesano, 49, 80131, Napoli, Italy; angela.corvino@unina.it (A.C.); antonia.scognamiglio@unina.it (A.S.); fefiorin@unina.it (F.Fi.); frecente@unina.it (F.Fr.); perissut@unina.it (E.P.); santagad@unina.it (V.S.); cirino@unina.it (G.Ci.); elisabetta.granato@unina.it (E.G.); ida.cerqua@unina.it (I.C.); roviezzo@unina.it (F.R.); caliendo@unina.it (G.Ca.)
[2]Department of Public Health, School of Medicine, University of Naples Federico II, Via Pansini, 5, 80131, Napoli, Italy; elisa.magli@unina.it (E.M.)
[3]Genetic S.p.A., Via della Monica, n. 26, 84083 Castel San Giorgio (SA) – Italy; rocco.pavese@geneticspa.com (R.P.); a.petti64@gmail.com (A.P.); francesca.pavese@geneticspa.com (F.Pa.); pettifrancesco95@gmail.com (F.Pe.)
*Correspondence: bseverin@unina.it (B.S.)

Abstract: In this study a new process for the preparation of budesonide 21-phosphate and its disodium salt is described. The obtained molecules are characterized by a better water solubility, compared to the parent. Moreover, they have been evaluated for their anti-inflammatory activity and the obtained results clearly evidence that budesonide 21-phosphate (Bud-21P) and budesonide 21-phosphate disodium salt (Bud-21P-Na2) retained anti-inflammatory activity like the parent compound budesonide (Bud) in mice with cutaneous induced edema.

Keywords: anti-inflammatory drugs; inhaled corticosteroids; inflammation; phosphorylation.

Table of content

Page S2……Figure S1. 1H-NMR (500 MHz; CD3OD-d4) spectrum of Budesonide 21-phosphate.
Page S3……Figure S2. 13C-NMR (126 MHz; CD3OD-d4) spectrum of Budesonide 21-phosphate.
Page S4……Figure S3. 1H-NMR (500 MHz; CD3OD-d4) spectrum of Budesonide 21-phosphate disodium salt.
Page S5……Figure S4. 13C-NMR (126 MHz; CD3OD-d4) spectrum of Budesonide 21-phosphate disodium salt.
Page S6……Figure S5. ESI-MS spectrum of Budesonide 21-phosphate.
Page S7……Figure S6. FT-IR spectrum of Budesonide 21-phosphate.
Page S8……Figure S7. FT-IR spectrum of Budesonide 21-phosphate disodium salt.

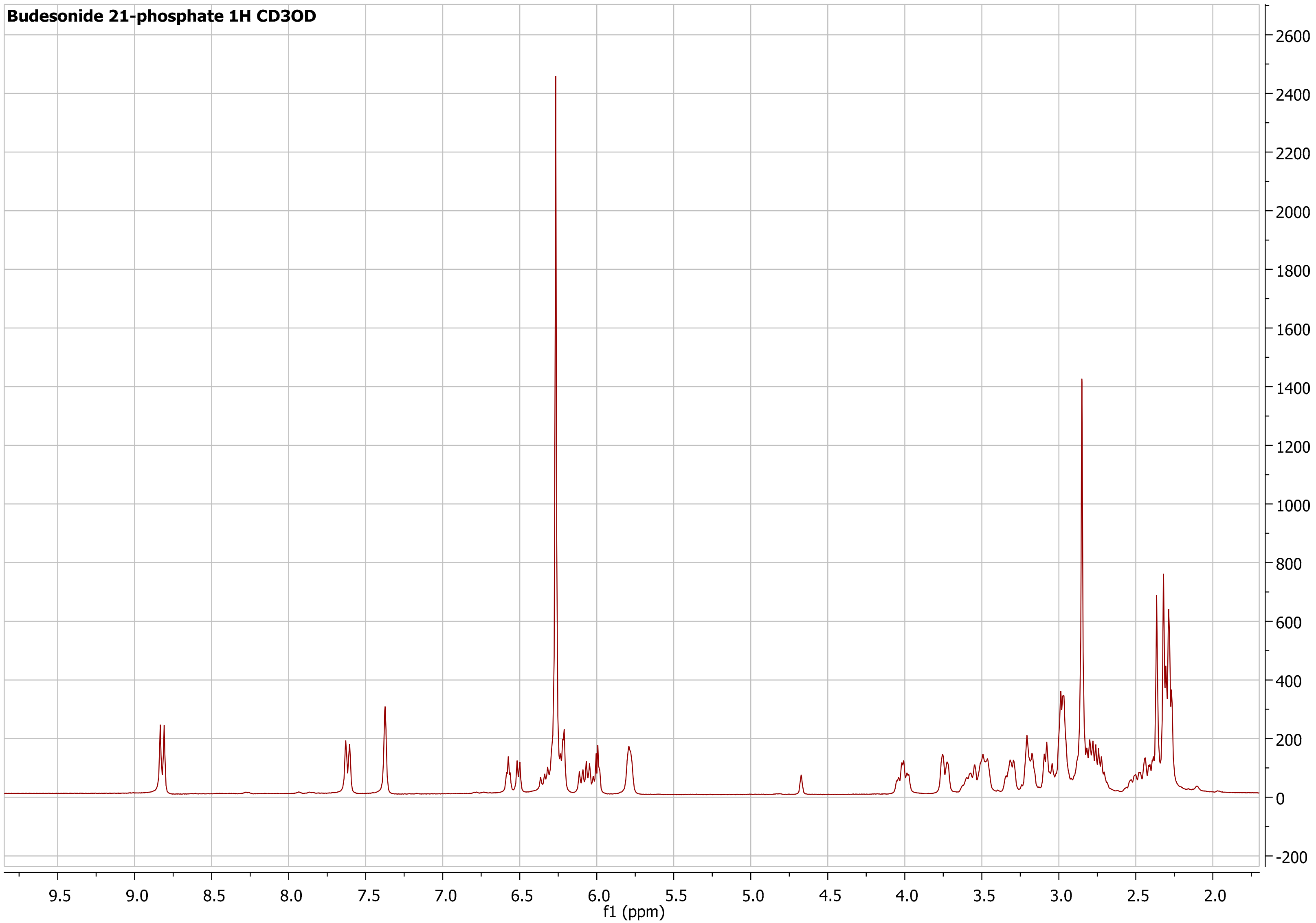

Figure S1. 1H-NMR (500 MHz; CD3OD-d4) spectrum of Budesonide 21-phosphate.

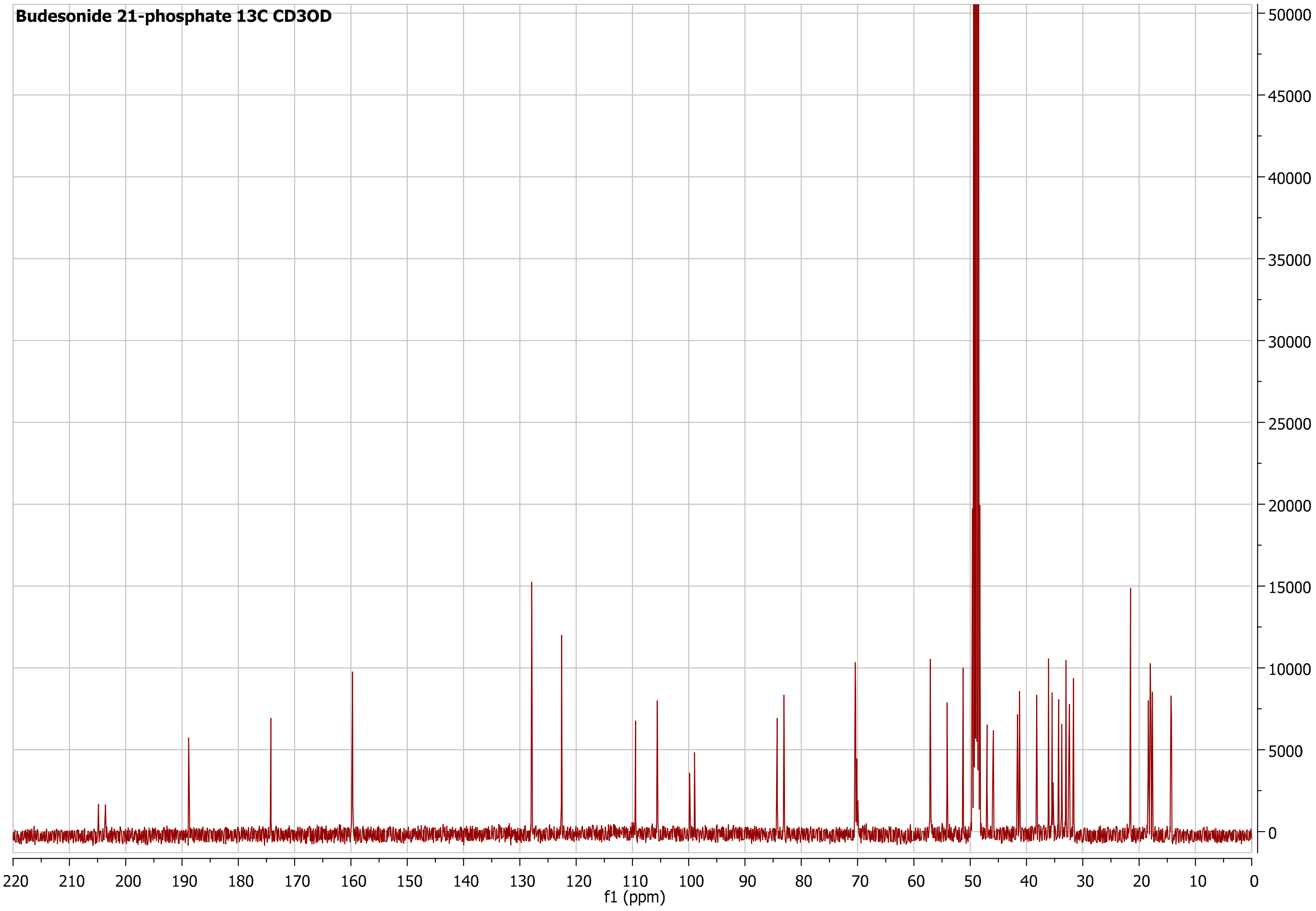

Figure S2. 13C-NMR (126 MHz; CD3OD-d4) spectrum of Budesonide 21-phosphate.

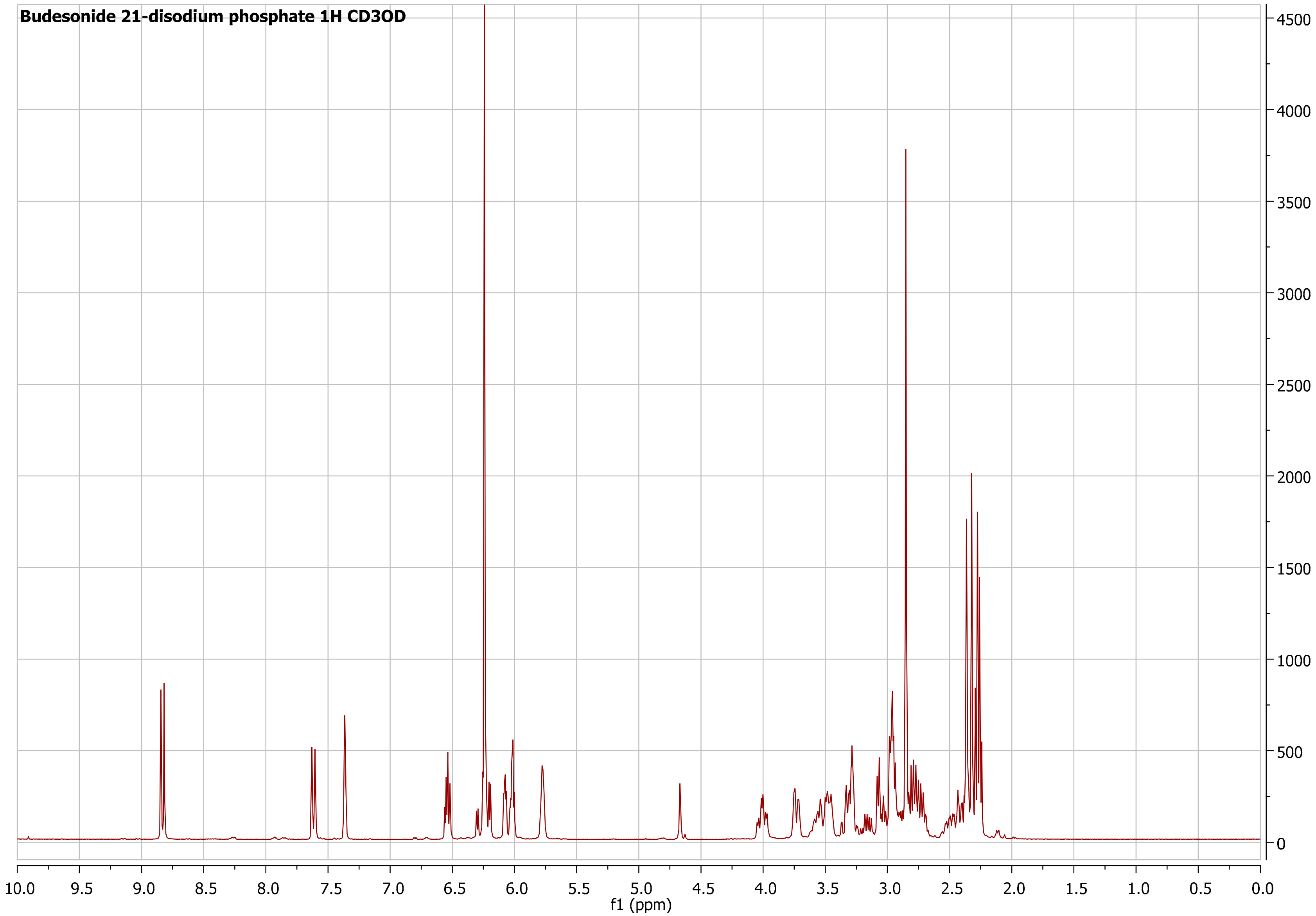

Figure S3. 1H-NMR (500 MHz; CD3OD-d4) spectrum of Budesonide 21-phosphate disodium salt.

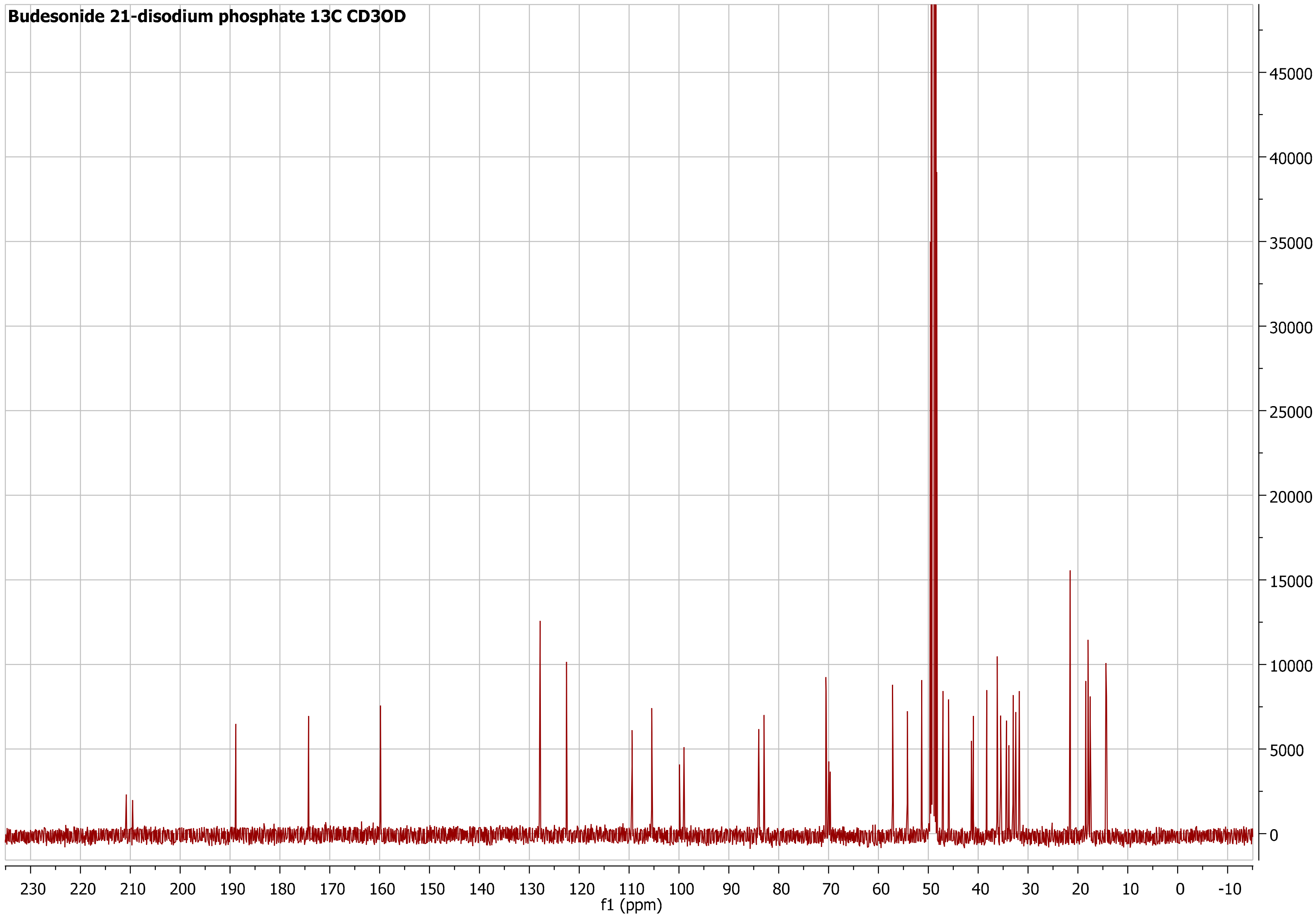

Figure S4. 13C-NMR (126 MHz; CD3OD-d4) spectrum of Budesonide 21-phosphate disodium salt.

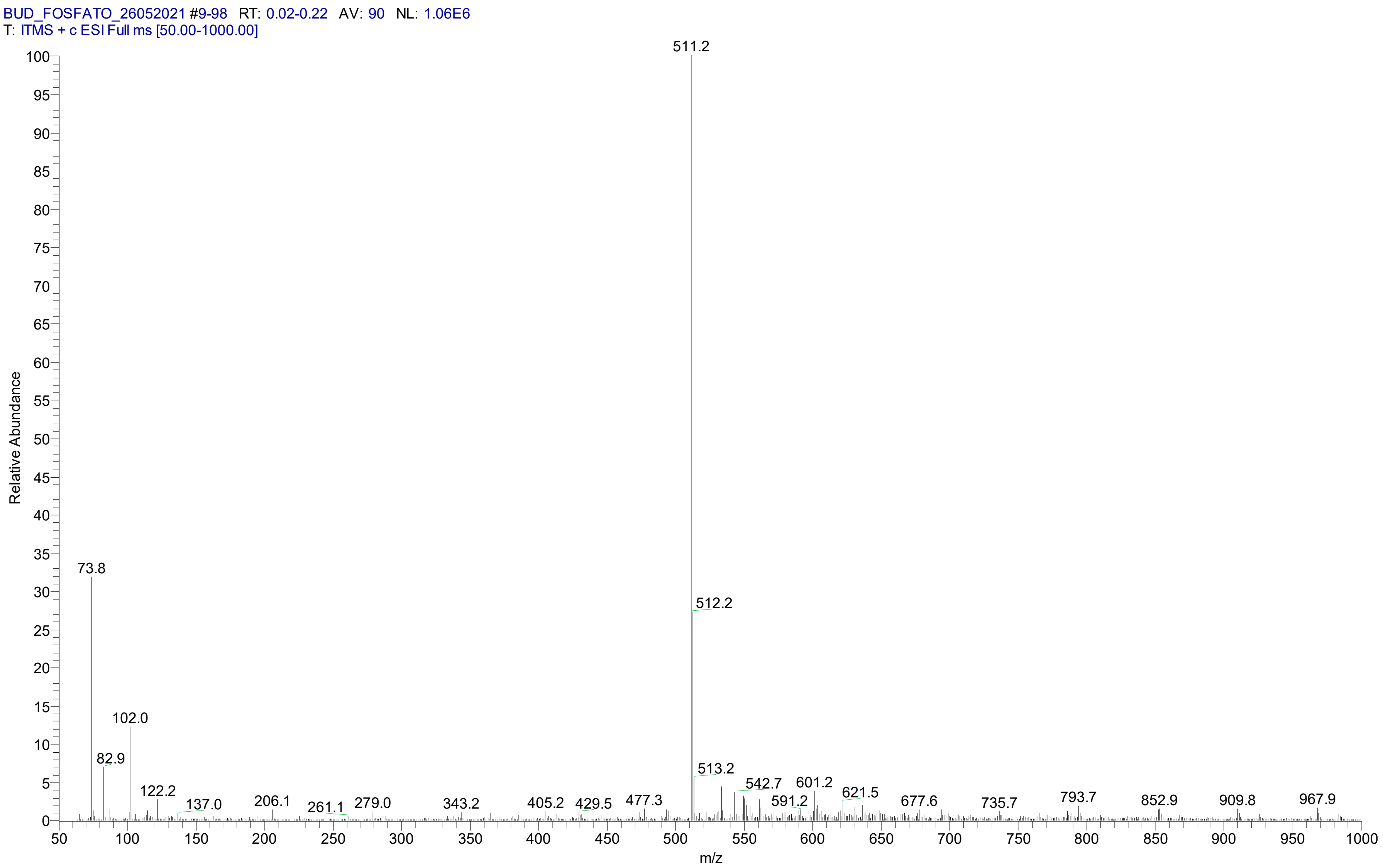

Figure S5. ESI-MS spectrum of Budesonide 21-phosphate.

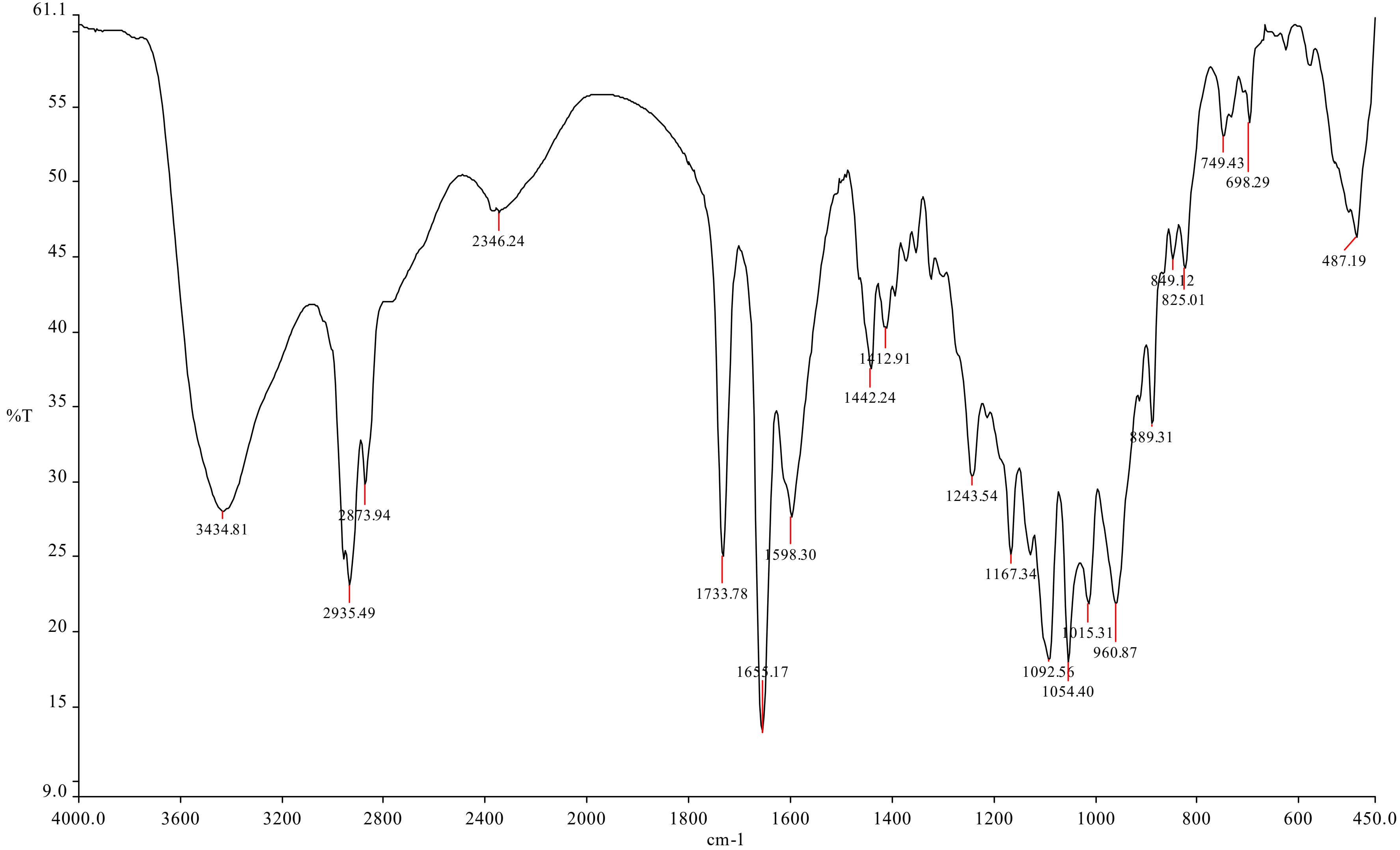

Figure S6. FT-IR spectrum of Budesonide 21-phosphate.

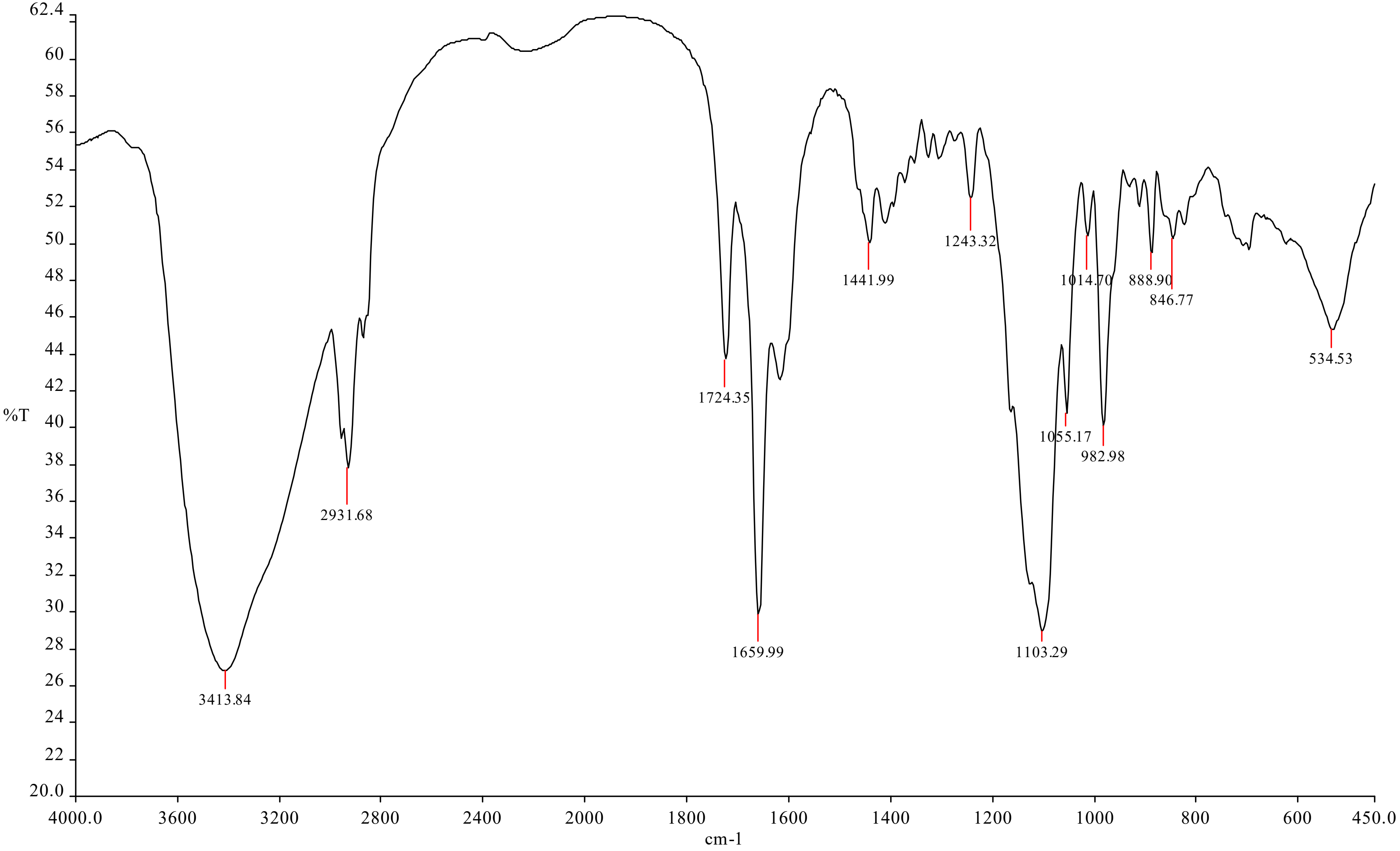

Figure S7. FT-IR spectrum of Budesonide 21-phosphate disodium salt.
